# Aldehyde Dehydrogenase 2 Lactylation Aggravates Mitochondrial Dysfunction by Disrupting PHB2 Mediated Mitophagy in Acute Kidney Injury

**DOI:** 10.1002/advs.202411943

**Published:** 2024-12-31

**Authors:** Jiaying Li, Xiaoxiao Shi, Jiatong Xu, Kaiyue Wang, Fangxing Hou, Xiaodong Luan, Limeng Chen

**Affiliations:** ^1^ Department of Nephrology State Key Laboratory of Complex Severe and Rare Diseases Peking Union Medical College Hospital Chinese Academy of Medical Science and Peking Union Medical College Beijing 100730 China; ^2^ Department of Internal Medicine Peking Union Medical College Hospital Chinese Academy of Medical Sciences and Peking Union Medical College Beijing 100730 China; ^3^ Center for Drug Research and Evaluation Institute of Clinical Medicine Peking Union Medical College Hospital Chinese Academy of Medical Science and Peking Union Medical College Beijing 100730 China

**Keywords:** acute kidney injury, ALHD2, lactylation, mitochondrial function, mitophagy

## Abstract

Mitochondrial dysfunction is a crucial event in acute kidney injury (AKI), leading to a metabolic shift toward glycolysis and increased lactate production. Lactylation, a posttranslational modification derived from lactate, plays a significant role in various cellular processes, yet its implications in AKI remain underexplored. Here, a marked increase in lactate levels and pan‐Kla levels are observed in kidney tissue from AKI patients and mice, with pronounced lactylation activity in injured proximal tubular cells identified by single‐cell RNA sequencing. The lactylation of aldehyde dehydrogenase 2 (ALDH2) is identified at lysine 52 (K52la), revealing that ALDH2 lactylation exacerbates tubular injury and mitochondrial dysfunction. Conversely, the ALDH2 K52R mutation alleviates these injuries in HK‐2 cells and adeno‐associated virus‐infected kidney tissues in mice. Furthermore, ALDH2 lactylation can be modulated by upregulating SIRT3 in vivo and in vitro, which reduces ALDH2 lactylation, mitigating tubular injury and mitochondrial dysfunction. Mechanistically, immunoprecipitation‐mass spectrometry analysis demonstrates an interaction between ALDH2 and prohibitin 2 (PHB2), a crucial mitophagy receptor. ALDH2 lactylation promotes the ubiquitination‐proteasomal degradation of PHB2 to inhibit mitophagy and worsen mitochondrial dysfunction. These findings highlight the critical role of endogenous lactate in AKI and propose ALDH2 lactylation as a potential therapeutic target.

## Graphical abstract

Lactylation of aldehyde dehydrogenase 2 (ALDH2) at lysine 52 (K52la) exacerbates tubular injury and mitochondrial dysfunction in acute kidney injury (AKI), which can be modulated by upregulating SIRT3. Mechanistically, ALDH2 lactylation promotes the ubiquitination‐proteasomal degradation of prohibitin 2 (PHB2), a crucial mitophagy receptor, thereby impairing mitophagy and aggravating mitochondrial dysfunction. These findings highlight the pathological role of endogenous lactate in AKI and propose ALDH2 lactylation as a promising therapeutic target.

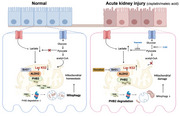

## 1 Introduction

Acute kidney injury (AKI) is a common clinical condition characterized by a rapid deterioration in renal function within a short period due to various reasons such as sepsis, trauma, cardiac surgery, nephrotoxic drugs, and its underlying chronic kidney disease (CKD), with significant morbidity and mortality, affecting over half of all patients in intensive care unit (ICU) worldwide.^[^ ^1^ ^]^ Contributing to most of the pathogenesis of AKI, the proximal tubular epithelial cells (PTCs), responsible for 90% of tubular reabsorption with their high mitochondrial density, are particularly susceptible to injury. Mitochondrial disorder plays a crucial role in AKI with reductions in mitochondrial quantity, structural deformations, and a decline in mitochondrial membrane potential, ultimately leading to cellular apoptosis and the progression toward chronic kidney disease (CKD).^[^ ^2^ ^]^

With mitochondrial dysfunction, enhanced aerobic glycolysis promotes lactate production, which is an independent predictor for the prognosis of patients with AKI.^[^ ^3^ ^]^ Recently, a novel posttranslational modification associated with lactate, known as lactylation, has been identified in both histone and non‐histone proteins.^[^ ^4^ ^]^ Lactylation plays a role in tumor progression, immunotherapy, and mitochondrial metabolism, and is regulated by various acylation writers such as p300, CBP, and GCN5, as well as erasers from the Sirtuin family.^[^ ^5^, ^6^ ^]^ A new study demonstrated that increased lactylation of mitochondrial fission 1 protein (Fis1 K20la) exacerbated mitochondrial dysfunction and fission in sepsis‐induced AKI, which could be alleviated by activating subunit alpha of PDH (PDHA1).^[^ ^7^ ^]^ However, the implications of lactylation on other proteins and its underlying mechanisms in AKI remain largely unexplored.

Therefore, investigating therapeutic targets to mitigate mitochondrial dysfunction in AKI is of paramount importance. Aldehyde dehydrogenase 2 (ALDH2) is a mitochondrial enzyme that metabolizes acetaldehyde into non‐toxic acetic acid, influencing mitochondrial oxidative ATP production and reactive oxygen species (ROS) in various diseases, such as myocardial,^[^ ^8^ ^]^ liver,^[^ ^9^ ^]^ and neurological disorders.^[^ ^10^ ^]^ Recently, more research has focused on the role of ALDH2 in renal pathologies, specifically in renal fibrosis, diabetic nephropathy, and AKI.^[^ ^11^, ^12^, ^13^ ^]^ Xu et al. demonstrated that ALDH2 could upregulate Beclin‐1 expression, enhance autophagy, and reduce apoptosis in murine models and human renal tubular epithelial cells exposed to Iohexol.^[^ ^14^ ^]^ Previously, we showed that ALDH2 suppressed glycolysis and lactate production, mitigating mitochondrial dysfunction by interacting with PGC‐1α (a key regulator of mitochondrial biogenesis) in AKI.^[^ ^15^ ^]^ However, the effects of ALDH2 lactylation and underlying role in regulating mitochondrial function in AKI have not yet been elucidated.

In this study, we report for the first time the specific lactylation site of ALDH2 and its effect on mitochondrial function both in vivo and in vitro. SIRT3 was identified as a delactylation enzyme for ALDH2, with its activator reducing ALDH2 lactylation and alleviating mitochondrial dysfunction. Mechanistically, immunoprecipitation‐mass spectrometry (IP‐MS) analysis revealed that ALDH2 interacted with prohibitin 2 (PHB2) and ALDH2 lactylation enhanced the proteasomal degradation of PHB2 and inhibited mitophagy, consequently exacerbating mitochondrial dysfunction. Hence, endogenous lactate and ALDH2 lactylation might be considered as potential therapeutic targets in patients with AKI.

## 2 Results

### 2.1 Elevated Lactate Levels Correlated with Renal Function Decline in AKI

**Figure 1:**
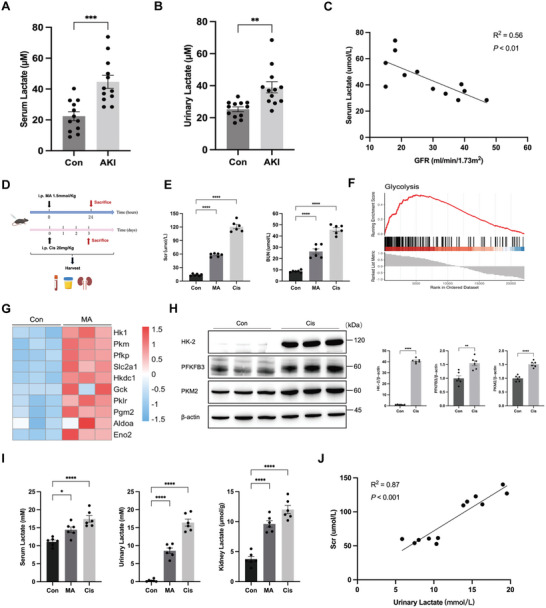
Correlation between elevated lactate levels and renal function decline in AKI patients and mice. A,B) The serum and urinary lactate levels were measured in control and AKI patients (n = 12). C) Correlation analysis between GFR and serum lactate levels in AKI patients. D) Schematic diagram of the cisplatin and MA‐AKI mice model. E) Serum creatinine (Scr) and urea nitrogen (BUN) levels in the Con, Cis‐AKI and MA‐AKI groups (n = 6). F,G) The gene set enrichment analysis (GSEA) and heatmap for differentially expressed genes (DEGs) from mRNA sequencing in Con and MA groups. H) The protein expression of aerobic glycolysis‐related enzymes (HK2, PFKFB3, and PKM2) was measured by Western blotting (n = 6). I) The lactate levels in serum, urine, and kidney tissues were measured in the Con, Cis‐AKI and MA‐AKI groups (n = 6). J) Correlation analysis between urinary lactate concentration and Scr in AKI mice. Unpaired student's t‐test, one‐way ANOVA and linear regression were used for the analysis. ^*^ *P* < 0.05, ^**^ *P* < 0.01, ^***^ *P* < 0.001, ^****^ *P* < 0.0001. (AKI, acute kidney injury; Con, control; Cis‐AKI, cisplatin‐induced AKI; MA‐AKI, maleic acid‐induced AKI).

Clinical evidence indicated that both serum and urine lactate levels in AKI patients were significantly higher than in the control group (**Figure** **1**A,B), with a negative correlation between serum lactate levels and glomerular filtration rate (GFR) among AKI patients (Figure 1C). Subsequently, the AKI mice model was induced by cisplatin and MA intraperitoneally (Cis‐AKI or MA‐AKI mice) with increased serum creatinine (Scr) and blood urea nitrogen (BUN) levels (Figure 1D,E). In addition to the upregulation of the glycolysis pathway and key genes observed in kidney tissues of MA‐AKI mice through mRNA sequencing (Figure 1F,G), Western blot analysis further confirmed a consistent increase in the protein expression of glycolysis‐related enzymes (HK‐2, PFKFB3, and PKM2) (Figure 1H). Lactate levels in serum, urine, and kidney tissues were significantly elevated in cisplatin and MA‐AKI mice compared to the control group, alongside a positive correlation between urinary lactate and Scr levels (Figure 1I,J), suggesting that lactate served as a critical biomarker in AKI.

### 2.2 Lactate Inhibition Attenuated MA‐Induced Renal Injury and Mitochondrial Dysfunction

**Figure 2:**
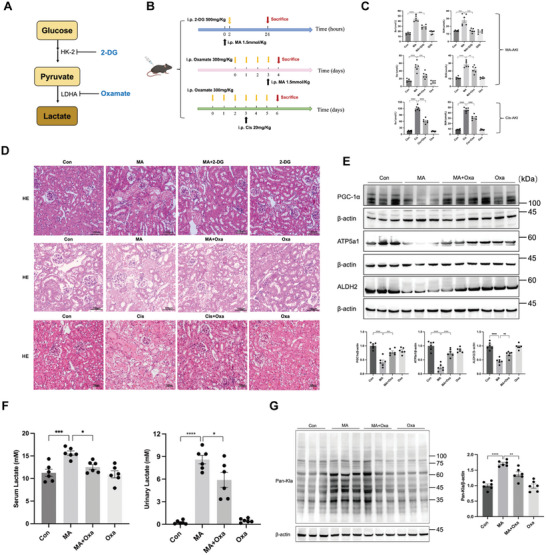
Inhibition of lactate production mitigated cisplatin and MA‐induced renal injury and mitochondrial dysfunction. A,B) Schematic diagram illustrating the mechanisms of inhibition of lactate production by 2‐DG and oxamate in vivo, and the experimental design of drug treatment for cisplatin‐ and MA‐AKI mice. C) Scr and BUN levels in 2‐DG and oxamate‐treated AKI mice (n = 6). D) Images of hematoxylin‐eosin (HE) staining (n = 6). Scale bars, 100µm. E) The expression of mitochondria‐related proteins (PGC‐1α, ATP5a1, and ALDH2) was measured by Western blotting (n = 6). F) The lactate levels in serum and urine were measured in oxamate‐pretreated MA‐AKI mice (n = 6). G) The levels of pan‐lactylation (Pan‐Kla) were measured by Western blotting (n = 6). One‐way ANOVA was used for the analysis. ^*^ *P* < 0.05, ^**^ *P* < 0.01, ^***^ *P* < 0.001, ^****^ *P* < 0.0001. (Con, control; MA, maleic acid; 2‐DG, 2‐Deoxy‐D‐glucose; Oxa, oxamate; MA‐AKI, maleic acid‐induced AKI).

Both 2‐DG and oxamate, inhibitors of the key enzymes in the glycolytic pathway, prevented increases in Scr and BUN in cisplatin and MA‐AKI mice (**Figure** **2**A–C) and abolished tubular necrosis partly in histopathological analysis (Figure 2D), with elevated levels of mitochondrial‐related proteins (PGC‐1α and ATP5a1) (Figure 2E; Figure S1C, S2A, Supporting Information). Additionally, 2‐DG mitigated the expression of kidney injury molecule‐1 (KIM‐1) and glycolysis‐associated enzymes (HK‐2 and PFKFB3) (Figure S1A,B, Supporting Information). Oxamate reduced the increased lactate content in serum and urine of MA‐AKI mice, alongside a similar trend in protein pan‐lactylation levels in kidney tissues (Figure 2F,G). These results indicated that blocking the glycolytic pathway of lactate production could effectively ameliorate tubular injury and mitochondrial dysfunction in MA‐AKI mice.

Lactate exposure in vitro exacerbated mitochondrial disorders of HK‐2 cells, evidenced by reduced expression of mitochondrial‐related proteins (PGC‐1α and ATP5a1), compromised mitochondrial membrane potential, and an increase in mitochondrial superoxide production (Figure S3A,C,D, Supporting Information). Additionally, the Seahorse assay revealed decreased baseline oxygen consumption rate (OCR) and diminished response to FCCP‐induced OCR elevation in lactate‐stimulated HK‐2 cells, indicating impaired mitochondrial respiration (Figure S3B, Supporting Information). Lactate further inhibited ALDH2 activity in MA‐treated HK‐2 cells (Figure S3E, Supporting Information).

### 2.3 Single‐Cell RNA Sequencing Revealed an Association Between Renal Lactylation and Proximal Tubular Injury

**Figure 3:**
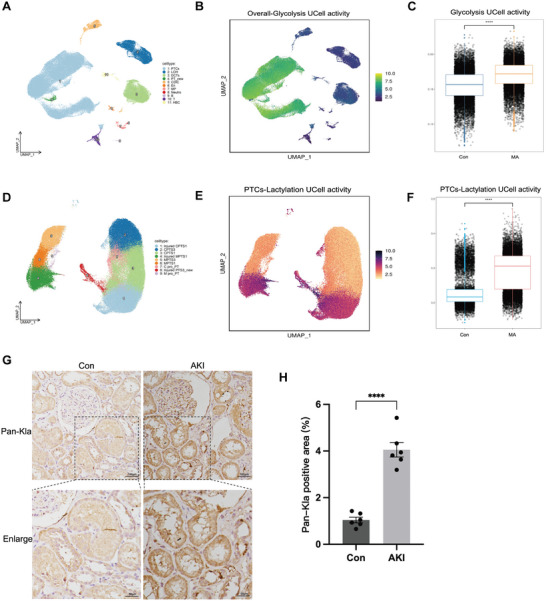
Association between renal lactylation and proximal tubular injury in MA‐AKI mice. A) UMAP plot visualization for all cell types of single‐cell sequencing. B,C) Glycolysis activity in different cell types and groups. D) UMAP plot visualization for PTCs clusters. E,F) Lactylation activity in different PTCs and groups. G,H) Levels and quantifications of Pan‐Kla in renal biopsy specimens from individuals with AKI and control participants detected by IHC staining. Scale bars, 100 µm (upper panels) and 50 µm (lower panels). Unpaired student's t‐test was used for the analysis. ^****^ *P* < 0.0001. (Con, control; MA, maleic acid; MA‐AKI, maleic acid‐induced AKI; PTCs, proximal tubule cells).

We conducted single‐cell RNA sequencing (scRNA‐seq) on kidney samples from both MA‐AKI and the control group, isolating a total of 79953 high‐quality cells across 11 distinct cell types, which included six types of renal parenchymal cells, four immune cell types, and red blood cells (**Figure** **3**A). Proximal tubular cells (PTCs) were re‐clustered and annotated using established biomarkers, revealing nine diverse states of PTCs (Figure 3D). Notably, glycolysis exhibited increased pathway activity in PTCs (Figure 3B), while lactylation activity was more pronounced in injured PTCs (Figure 3E). Both pathways showed higher activity levels in the MA‐AKI group than in the control group (Figure 3C,F), confirming the essential role of lactylation in proximal tubular injury. Based on immunohistochemical (IHC) analysis, renal tissues from patients with AKI exhibited significantly elevated levels of Pan‐Kla compared to those with minor lesions (Figure 3G,H).

### 2.4 ALDH2 Lactylation was Crucial in Regulating Mitochondrial Function

**Figure 4:**
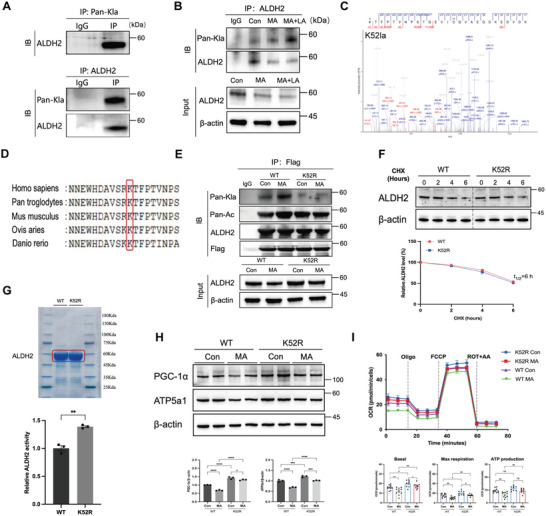
The lactylation of ALDH2 in AKI was crucial in regulating mitochondrial function. A,B) ALDH2 lactylation was increased in MA and lactate‐stimulated HK‐2 cells. C) Illustration of possible lactylation sites of ALDH2 analyzed via immunoprecipitation (IP)‐mass spectrometry (MS) analysis. D) The sequences around ALDH2 K52 were conserved from different mammalian species. Conserved lysine residues were marked in red. E) ALDH2 lactylation and acetylation levels in different groups were measured by Western blotting (n = 3). F) Cycloheximide (CHX) chase assay for the half‐time life of ALDH2 in HK‐2 cells treated with CHX (100 µg ml^−1^) for the indicated time points (n = 3). G) ALDH2 activity was measured in the WT and K52R groups with equal amounts of protein (n = 3). H) The expression of mitochondria‐related proteins (PGC‐1α and ATP5a1) was measured by Western blotting (n = 3). I) Measurement of mitochondrial oxygen consumption ratio (OCR) in different groups (n = 9–11 for each group). Unpaired student's t‐test and two‐way ANOVA were used for the analysis. ^*^ *P* < 0.05, ^**^ *P* < 0.01, ^***^ *P* < 0.001, ^****^ *P* < 0.0001. (Con, control; MA, maleic acid; LA, lactate; WT, wild type; CHX, cycloheximide; OCR, oxygen consumption ratio; Oligo, oligomycin; ROT+AA, rotenone/antimycin A).

ALDH2 is known to maintain mitochondrial homeostasis in AKI^,^ but the effects of its lactylation have not been elucidated. In our study, immunoprecipitation (IP) revealed a significant increase in ALDH2 lactylation following treatment with MA and lactate (**Figure** **4**A,B). IP‐MS analysis identified a single lactylated lysine residue on ALDH2 (K52), which is relatively conserved across species (Figure 4C,D). To further investigate the impact of K52la on ALDH2 and mitochondrial function, Flag‐ALDH2 (wild‐type [WT]) and Flag‐ALDH2‐K52R plasmids were transfected into MA‐treated HK‐2 cells. Both mRNA and protein expression levels of ALDH2 were increased in cells transfected with either ALDH2‐WT or ALDH2‐K52R plasmids compared to the control group (Figure S4A,B, Supporting Information), indicating successful transfection. The K52R mutation notably reduced ALDH2 lactylation levels and increased its enzymatic activity (1.39±0.02 vs 1.01±0.05 umol L^−1^, *P* < 0.01) based on the equal amounts of protein, without affecting ALDH2 protein expression (Figure 4E–G). In addition, the mitochondrial‐related proteins (PGC‐1α and ATP5a1) and mitochondrial respiration capacity measured by Seahorse were improved in K52R‐transfected cells (Figure 4H,I), suggesting that ALDH2 lactylation primarily occurred at the K52 site, affecting ALDH2 activity and mitochondrial function.

### 2.5 AAV‐Mediated ALDH2 K54R Overexpression Ameliorated Tubular Injury and Mitochondrial Dysfunction in MA‐AKI Mice

**Figure 5:**
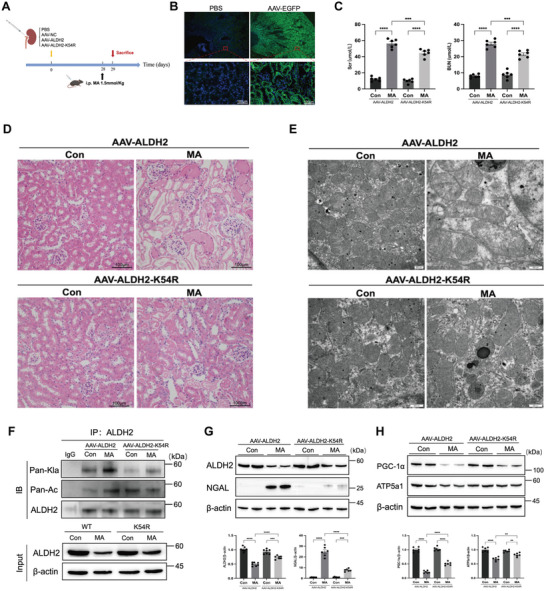
The effect of AAV‐ALDH2‐K54R on ALDH2 lactylation, renal injury, and mitochondrial dysfunction in MA‐induced AKI mice. Mice were initially injected with AAV9‐ALDH2‐EGFP or AAV9‐ALDH2‐K54R‐EGFP into the renal pelvis, followed 28 days later by a single MA injection. A) The flowchart of mice modeling. B) Fluorescence images of AAV‐EGFP in cryopreserved kidney tissue sections were used to assess AAV infectivity. Scale bars, 100µm. C) Serum creatinine (Scr) and urea nitrogen (BUN) levels in different groups (n = 6). D) Images of hematoxylin‐eosin (HE) staining (n = 6). Scale bars, 100µm. E) Representative transmission electron microscopy (TEM) micrographs of mouse renal tubular epithelial cell mitochondria in each group. Scale bars, 0 .5µm. F) The levels of pan‐lactylation (Pan‐Kla) protein were measured by Western blotting (n = 3). G) The expression of ALDH2 and NGAL was measured by Western blotting (n = 6). H) The expression of mitochondria‐related proteins (PGC‐1α and ATP5a1) was measured by Western blotting (n = 6). Two‐way ANOVA was used for the analysis. ^*^ *P* < 0.05, ^**^ *P* < 0.01, ^***^ *P* < 0.001, ^****^ *P* < 0.0001. (Con, control; MA, maleic acid; AAV, adeno‐associated viruses; NC, normal control).

To verify the function of the ALDH2 lactylation site, mice were injected with AAV9‐ALDH2‐EGFP or AAV9‐ALDH2‐K54R‐EGFP via renal pelvis (**Figure** **5**A). Luminescence imaging showed predominant expression in the left kidney, with the minimal signal detected in the right kidney (Figure S5A, Supporting Information). The immunofluorescent technique revealed efficient kidney transduction in mice injected with AAV‐EGFP compared to PBS control (Figure 5B). Mice treated with PBS, AAV‐NC, and AAV‐ALDH2 exhibited comparable renal function and pathology structures without significant changes in NGAL protein levels, except for increased ALDH2 expression in the AAV‐ALDH2 group (Figure S5B–D, Supporting Information). These results indicated the safety of AAV9‐mediated ALDH2 overexpression in the kidney. Furthermore, mice were administrated MA 28 days following the AAV injection. The results demonstrated that AAV‐mediated overexpression of ALDH2‐K54R significantly reduced Scr (56.35±5.43 vs 44.68±4.65 µmol L^−1^, *P* = 0.0003) and BUN (27.97±2.47 vs 21.50±2.24 µmol L^−1^, *P* = 0.0002) levels compared to the ALDH2‐WT group and attenuated the extent of renal tubular injury as well as mitochondrial swelling and vacuolation in MA‐AKI mice (Figure 5C–E). The Western blot analysis revealed decreased levels of ALDH2 lactylation and NGAL expression, with a partial restoration of mitochondrial‐related proteins in AAV‐ALDH2‐K54R group (PGC‐1α and ATP5a1) (Figure 5F–H).

### 2.6 SIRT3 Affected the Lactylation of ALDH2 in Regulating Mitochondrial Function

**Figure 6:**
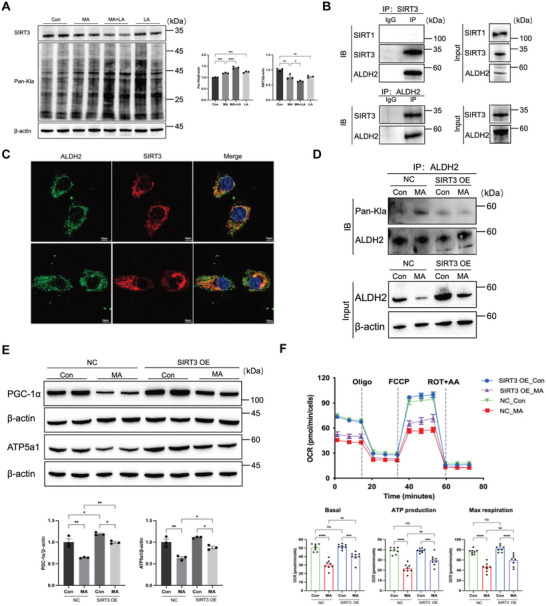
SIRT3 served as a lactylation eraser and affected the lactylation of ALDH2. A) The levels of pan‐lactylation (Pan‐Kla) protein and SIRT3 in MA and lactate‐stimulated HK‐2 cells (n = 3). B) Co‐immunoprecipitation assay showed the interaction of ALDH2 with SIRT3 (not Sirt1) in HK‐2 cells (n = 3). Input immunoblotted is shown as a control. C) Immunofluorescent analysis of the location of ALDH2 and SIRT3 in HK‐2 cells (n = 3). D) The level of ALDH2 lactylation was measured by Western blotting in SIRT3‐overexpressing HK‐2 cells (n = 3). E) The expression of mitochondria‐related proteins (PGC‐1α and ATP5a1) was measured by Western blotting in SIRT3‐overexpressing HK‐2 cells (n = 3). F) Measurement of mitochondrial oxygen consumption ratio (OCR) in SIRT3‐overexpressing HK‐2 cells (n = 8). One‐way and two‐way ANOVA were used for the analysis. ^*^ *P* < 0.05, ^**^ *P* < 0.01, ^***^ *P* < 0.001, ^****^ *P* < 0.0001. (Con, control; MA, maleic acid; LA, lactate; NC, normal control; OE, overexpression; OCR, oxygen consumption ratio; Oligo, oligomycin; ROT+AA, rotenone/antimycin A).

Lactylation is a newly recognized form of lysine acylation, potentially sharing similar writers and erasers with other acylation types. Considering the critical role of Sirtuin family proteins as deacetylases, we selected SIRT1 and SIRT3 as candidate enzymes for delactylating ALDH2. We observed elevated pan‐lactylation levels in HK‐2 cells treated with MA and lactate, while SIRT3 expression decreased (**Figure** **6**A), suggesting that SIRT3 might act as a lactylation eraser. Co‐immunoprecipitation confirmed the interaction between SIRT3 and ALDH2, which was further supported by colocalization of double‐label immunofluorescence (Figure 6B,C). To elucidate the role of SIRT3 in regulating lactylation, we overexpressed SIRT3 in MA‐treated HK‐2 cells (transfection efficiency was evaluated in Figure S4C,D, Supporting Information), resulting in a notable reduction in ALDH2 lactylation levels (Figure 6D). Concurrently, the mitochondrial‐related proteins (PGC‐1α and ATP5a1), as well as mitochondrial respiration capacity, mitochondrial membrane potential, and mitochondrial superoxide were partially recovered in cells with SIRT3 overexpression (Figure 6E,F; Figure S6A,B, Supporting Information).

**Figure 7:**
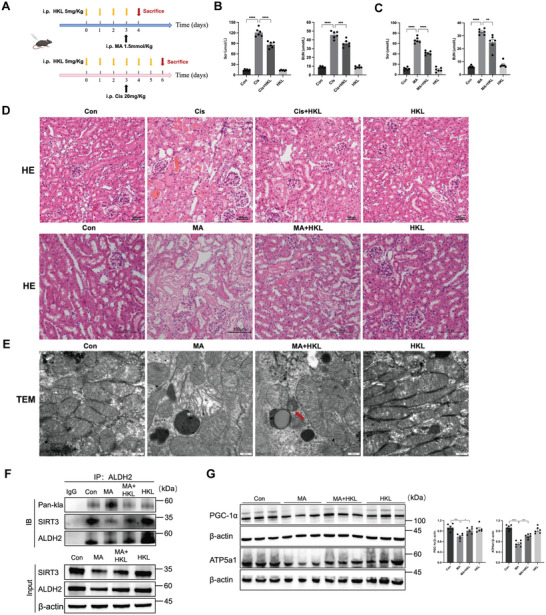
SIRT3 activation attenuated renal injury and mitochondrial dysfunction in cisplatin and MA‐induced AKI. A) Honokiol (SIRT3 activator, 5 mg k^−1^g) was injected intraperitoneally (i.p.) for three days before the injection of ciplatin and MA. B,C) Serum creatinine (Scr) and urea nitrogen (BUN) levels in cisplatin‐ and MA‐AKI mice (n = 6). D) Images of hematoxylin‐eosin (HE) staining (n = 6). Scale bars, 100µm. E) Representative transmission electron microscopy (TEM) micrographs of mouse renal tubular epithelial cell mitochondria in each group. The red arrow showed autophagosomes. Scale bars, 0 .5µm. F) The level of ALDH2 lactylation was measured by Western blotting (n = 6). G) The expression of mitochondria‐related proteins (PGC‐1α and ATP5a1) was measured by Western blotting (n = 6). One‐way ANOVA was used for the analysis. ^*^ *P* < 0.05, ^**^ *P* < 0.01, ^***^ *P* < 0.001, ^****^ *P* < 0.0001. (Con, control; MA, maleic acid; HKL, honokiol).

Following activating SIRT3 with honokiol (HKL) treatment, we observed improved renal function and decreased tubular necrosis in cisplatin and MA‐AKI mice (**Figure** **7**A–D). TEM results showed HKL ameliorated mitochondrial structure damage, accompanied by the appearance of autophagosomes (Figure 7E). The ALDH2 lactylation levels decreased after administration of HKL, concomitant with enhanced expression of mitochondrial‐related proteins (PGC‐1α and ATP5a1) (Figure 7F,G; Figure S2B, Supporting Information). These findings implied that ALDH2 delactylation exerted a protective role on mitochondrial dysfunction in AKI, potentially facilitated by the activation of autophagy.

### 2.7 ALDH2 K52 Lactylation Diminished ALDH2‐PHB2 Interaction and Impaired Mitophagy

**Figure 8:**
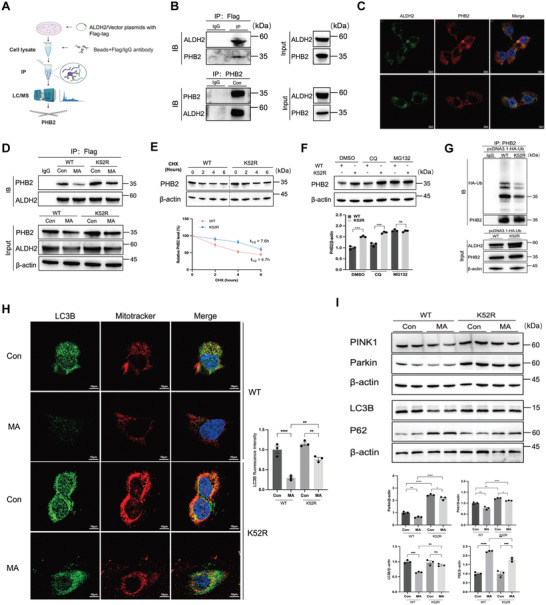
ALDH2 K52 lactylation reduced ALDH2‐PHB2 interaction and inhibited mitophagy. A) Schematic diagram illustrating the immunoprecipitation (IP) ‐mass spectrometry (MS) analysis for the detecting of proteins interacting with ALDH2. B) Co‐immunoprecipitation assay showed the interaction of ALDH2 with PHB2 in HK‐2 cells (n = 3). Input immunoblotted is shown as a control. C) Immunofluorescent analysis of the location of ALDH2 and PHB2 in HK‐2 cells (n = 3). D) In HK‐2 cells transfected with ALDH2‐WT and ALDH2‐K52R plasmids, the expression of ALDH2 and PHB2 was measured by Western blotting (n = 3). E) Cycloheximide (CHX) chase assay for PHB2 in HK‐2 cells treated with CHX (100 µg ml^−1^) for the indicated time points (n = 3). F) The expression of PHB2 in HK‐2 cells treated with 10µM CQ or 10µM MG132 for 6 h (n = 3). G) Co‐immunoprecipitation assay for the ubiquitination of PHB2 in HEK‐293T cells transfected with HA‐tagged ubiquitin, ALDH2‐WT, and ALDH2‐K52R plasmids (n = 3). H) The localization of LC3B and mitochondria was analyzed by immunofluorescence staining in different groups. Scale bars, 10µm. I) The expression of mitophagy‐related proteins (Pink1, Parkin, LC3B and P62) was measured by Western blotting (n = 3). Unpaired student's t‐test and two‐way ANOVA were used for the analysis. ^*^ *P* < 0.05, ^**^ *P* < 0.01, ^***^ *P* < 0.001, ^****^ *P* < 0.0001. (Con, control; MA, maleic acid; CQ, chloroquine; DMSO, dimethyl sulfoxide; CHX, cycloheximide; Ub, ubiquitination).

To investigate the mechanisms by which ALDH2 lactylation modulated the mitochondrial homeostasis of PTCs, we employed LC‐MS/MS to analyze immunoprecipitants from HK‐2 cells transfected with either Flag‐ALDH2 or control vector plasmids (**Figure** **8**A). This analysis identified 30 potential interacting proteins with coverage sequences exceeding 10% and possessing more than five unique peptides (Table S4, Supporting Information). Among them, prohibitin 2 (PHB2), an inner mitochondrial membrane protein, emerged as a significant mitophagy receptor in mammalian cells and C. elegans.^[^ ^16^ ^]^ Co‐immunoprecipitation assays confirmed the interaction between ALDH2 and PHB2, with colocalization within HK‐2 cells by immunofluorescence (Figure 8B,C). Additionally, K52R‐transfected cells showed increased PHB2 protein expression (Figure 8D) without significant changes in mRNA levels (Figure S7A, Supporting Information). The cycloheximide (CHX) chase assay demonstrated that the K52R mutation in ALDH2 extended the half‐life of the PHB2 protein in HK‐2 cells (7.6 h vs 4.7 h) (Figure 8E). In addition, the proteasome inhibitor MG132, but not the autophagy inhibitor chloroquine (CQ), abolished the PHB2 upregulation induced by K52R mutation (Figure 8F), indicating that ALDH2 lactylation promoted the proteasomal degradation of PHB2. This was further corroborated by decreased PHB2 ubiquitination in HEK‐293T cells transfected with ALDH2‐K52R and HA‐Ub plasmids (Figure 8G).

The colocalization of LC3 with Mitotracker, an established marker of mitophagy, was more pronounced in the K52R mutation groups (Figure 8H). The K52R mutation upregulated the expression of mitophagy‐associated proteins (Pink1, Parkin, LC3B, and P62) in MA‐treated HK‐2 cells (Figure 8I). Additionally, HKL also promoted mitophagy in MA‐AKI mice (Figure S6C, Supporting Information). To verify the involvement of PHB2 in regulating the effects of ALDH2 lactylation on mitochondrial function, HK‐2 cells were transfected with siPHB2 before introducing WT or K52R plasmids. PHB2 knockdown nearly eliminated the beneficial effects of K52R on mitochondrial dysfunction, as indicated by the decreased mitochondria‐related proteins (Figure S7B, Supporting Information). These results demonstrated that ALDH2 lactylation exacerbated tubular injury and mitochondrial dysfunction in AKI, potentially induced by disturbances in PHB2‐mediated mitophagy.

## 3 Discussion

Mitochondrial dysfunction of proximal tubule cells plays a crucial role in the pathogenesis of AKI, which disrupts normal cellular metabolism by shifting energy production from oxidative phosphorylation (OXPHOS) to less efficient glycolysis, leading to lactate accumulation.^[^ ^17^ ^]^ Our previous study confirmed the essential effect of ALDH2 on glycolysis and mitochondrial function through its interaction with peroxisome proliferator‐activated receptor gamma coactivator 1‐alpha (PGC‐1α).^[^ ^15^ ^]^ However, the effects of lactate and lactylation on ALDH2 and mitochondrial homeostasis in AKI remain unclear. This study revealed the pivotal role of ALDH2 lactylation in mitochondrial function during AKI progression by identifying its specific lactylation site and delactylation enzyme (SIRT3) both in vivo and in vitro. Mechanistically, ALDH2 lactylation impaired mitophagy by reducing interaction with PHB2, promoting its proteasomal‐ubiquitination degradation, further aggravating mitochondrial dysfunction.

Previous studies have confirmed that elevated lactate was an independent predictor for the prognosis of AKI patients, accompanied by local acidosis, exacerbating mitochondrial dysfunction and further impairing cellular metabolism.^[^ ^18^, ^19^ ^]^ Our study observed that lactate aggravated mitochondrial dysfunction and suppressed ALDH2 activity in HK‐2 cells. Inhibiting lactate production with glycolytic inhibitors (2‐DG) or lactate dehydrogenase (LDH) inhibitor (oxamate) mitigated tubular injury and mitochondrial dysfunction in AKI mice, in line with findings from other research.^[^ ^7^, ^20^, ^21^, ^22^ ^]^

Lactylation, a post‐translational modification derived from lactate, plays a significant role in mitochondrial function by modulating key metabolic enzymes and mitochondrial dynamics. Hypoxia induced mitochondrial alanyl‐tRNA synthetase (AARS2) accumulation, leading to lactylation of PDHA1 and carnitine palmitoyltransferase 2 (CPT2), which inactivated both enzymes and inhibited oxidative OXPHOS by limiting acetyl‐CoA influx from pyruvate and fatty acid oxidation.^[^ ^23^ ^]^ Additionally, increased lactylation of histone and mitochondrial fission protein 1 (Fis1) led to mitochondrial fragmentation and cellular apoptosis in LPS‐induced AKI as a potential therapy target.^[^ ^7^, ^24^ ^]^ In our study, significantly increased pan‐Kla levels in kidney tissue and lactylation activity in injured PTCs by scRNA‐seq indicated the indispensable role of lactylation in the progression of AKI. Subsequently, we observed the lactylation on ALDH2 at lysine 52 (K52la). This site, previously known for acetylation,^[^ ^25^ ^]^ had not been associated with lactylation before. In our study, the K52R mutation notably reduced ALDH2 lactylation levels, enhanced its activity without affecting ALDH2 acetylation significantly, and alleviated tubular injury and mitochondrial disorders in HK‐2 cells and AAV‐infected kidney tissues in mice, marking the first study of ALDH2 lactylation and its protective effects on mitochondrial function. Our findings demonstrated that ALDH2 lactylation at K52R played a critical role independently of acetylation, suggesting that targeting this specific modification could offer novel therapeutic insights.

Lactylation, similar to acylation, can be directly regulated by specific writers and erasers, such as SIRT3, a well‐known mitochondrial deacetylase, recently identified as an eraser of lactylation.^[^ ^26^ ^]^ It can reverse lactylation on crucial mitochondrial enzymes such as PDHA1 and CPT2, reactivating these enzymes and enhancing OXPHOS.^[^ ^23^ ^]^ In our study, we overexpressed SIRT3 in HK‐2 cells and activated SIRT3 with honokiol in cisplatin and MA‐AKI mice, observing reduced ALDH2 lactylation and mitigated mitochondrial dysfunction, which first indicated SIRT3 could act as an eraser of ALDH2 lactylation. However, further exploration is required to uncover the interplay between ALDH2 lactylation and acetylation.

The most impressive finding of our study is that ALDH2 lactylation impaired mitophagy by diminishing interaction with PHB2, which promoted its degradation by ubiquitin‐proteasome system. PHB2, a highly conserved protein in the inner mitochondrial membrane, is significant for mitophagy as it binds to LC3 and interacts with components of the PINK1‐Parkin pathway, promoting the selective removal of damaged mitochondria and maintaining mitochondrial quality control.^[^ ^27^, ^28^, ^29^, ^30^ ^]^ The retention of PHB2 within mitochondria is critical for maintaining renal tubular function, underscoring its role in mitophagy and mitochondrial integrity.^[^ ^31^ ^]^ Our IP‐MS analysis showed that ALDH2 interacted with PHB2, and we first demonstrated that ALDH2 lactylation disrupted PHB2 stabilization in HK‐2 cells via ubiquitin‐proteasomal mediated degradation. Although ALDH2 could increase K48‐linked polyubiquitination and degradation of certain proteins,^[^ ^32^ ^]^ the polyubiquitination involved in ALDH2 lactylaiton‐mediated PHB2 degradation asked for further investigation.

Although our study demonstrated the effects of ALDH2 K52la on mitochondrial dysfunction in vivo and in vitro, there are still some limitations. First, employing more sensitive approaches, such as lactylation‐specific antibody enrichment, could facilitate the detection of low‐abundance or novel lactylation sites on ALDH2. Second, a specific ALDH2–K52la antibody or knock‐out mice is needed to confirm these findings. Third, the role of other Sirtuin family proteins in delactylation should be evaluated, and the interaction between acetylation and lactylation requires further investigation.

In conclusion, our study revealed that increased lactate levels and ALDH2 lactylation at lysine 52 (K52la) promoted mitochondrial dysfunction in AKI, mediated by a reduced interaction with PHB2. These findings shed light on targeting lactate and ALDH2 lactylation as a potential strategy for AKI.

## 4 Experimental Section

### Human Serum and Urine Samples

The serum and urine samples were collected from patients with minor lesions (n = 12) and AKI (n = 12), confirmed by renal pathology, with the approval of the Ethics Committee of Peking Union Medical College Hospital (ethics approval no. I‐22PJ856) and the written informed consent. The characteristics of all participants were in Table S1 (Supporting Information).

### Animals and Treatments

Male C57BL/6 mice (19–23 g, 7–8 weeks old) were procured from Beijing Vital River Laboratory Animal Technology Company and maintained in a specific pathogen‐free (SPF) environment. The mice were randomly allocated into groups (n = 6 per group). All experimental procedures were approved by the Peking Union Medical College Hospital Institutional Ethics Committee of Animal Care and Use (No. XHDW‐2023‐171) and adhered to the National Institutes of Health Guide for the Care and Use of Laboratory Animals. The cisplatin (Cis‐AKI) and maleic acid‐induced acute kidney injury (MA‐AKI) was established model as described before,^[^ ^15^ ^]^ with 2‐Deoxy‐D‐glucose (2‐DG; 500 mg k^−1^g, Selleck, USA) administered intraperitoneally 0.5 hours post‐MA injection. Oxamate (300 mg k^−1^g, Selleck, USA) and honokiol (5 mg k^−1^g, MCE, USA) were administered intraperitoneally three days before cisplatin or MA injection. Following euthanasia, kidneys and blood samples were rapidly collected and serum creatinine (Scr) and blood urea nitrogen (BUN) levels were quantified with commercial assay kits (Jiancheng Biotech, China).

### Renal Pelvic Delivery of ALDH2 Gene using AAV9 Viral Vector

To achieve overexpression of ALDH2 in renal tubules, a cDNA encoding the ALDH2 sequence (wild‐type or K54R), an enhanced GFP reporter gene, and the tubular‐specific KSP promoter were constructed and inserted into an adeno‐associated virus 9 (AAV9) packaging vector (AAV9‐Ksp‐cadherin p‐MCS‐3flag‐T2A‐EGFP, produced by Shanghai Genechem Co., Ltd). Mice were randomly assigned to receive the wild‐type or K54R mutant ALDH2 gene. Following anesthesia with tribromoethanol via intraperitoneal injection, the left kidney was exposed, and 50 µL of AAV (1.5×10¹^2^ v.g. ml^−1^) containing AAV9‐ALDH2‐EGFP or AAV9‐ALDH2‐K54R‐EGFP constructs were gently injected through the renal pelvis using an insulin syringe, followed by slow withdrawal of the syringe and compression of the injection site with a cotton swab for 3–5 minutes. The kidney was then returned to the abdominal cavity, closed the wound with sutures, and maintained the animals on a heating pad until they recovered. Additionally, a control group received PBS via renal pelvic injections. Twenty‐eight days post‐AAV injection, maleic acid intraperitoneally was administered to induce AKI as previously described.

### In Vivo Bioluminescence Imaging

The AAV‐treated mice received an intraperitoneal injection of 100 µL D‐Luciferin (15 mg mL^−1^; Solarbio, China). Bioluminescence images were captured using the PerkinElmer IVIS Spectrum system five minutes post D‐Luciferin administration. Luminescence measurements were acquired and analyzed using Living Image software.

### Cell Culture and Treatments

The immortalized human proximal tubular epithelial cell line (HK‐2) was from Procell Life Science & Technology Company (CL‐0109, China). Cells were cultured in DMEM‐F12 medium supplemented with 10% fetal bovine serum and 1% penicillin‐streptomycin at 37 °C in a 5% CO₂ environment. HK‐2 cells were transfected with Flag‐ALDH2, Flag‐ALDH2‐K52R, and Flag‐SIRT3 plasmids (synthesized by Beijing SYKM Gene Biotechnology Co., Ltd.) using Lipofectamine 3000 (Invitrogen, USA) for 24 hours, then exposed to 1 mM maleic acid (MA; Sigma Aldrich, USA) with or without L‐lactic acid (MCE, USA) for 24 hours. Small interfering RNA (siRNA) targeting PHB2 (siPHB2), obtained from Guangzhou RiboBio Co., Ltd., was transfected into HK‐2 cells using RNAiMAX (Invitrogen, USA). To inhibit autophagy and proteasome activity, The HK‐2 cells were treated with chloroquine (10 µM; MCE, USA), MG132 (10 µM; MCE, USA), or dimethyl sulfoxide (DMSO) for 6 hours.

### Histological Examination and Immunohistochemical Staining

Kidney tissues were fixed in 4% paraformaldehyde for 24 hours and subsequently embedded in paraffin. Paraffin sections (4 µm) were deparaffinized, rehydrated, and stained with hematoxylin and eosin (HE). For immunohistochemical (IHC) staining, kidney tissue sections underwent antigen retrieval using citrate buffer (pH 6.0), followed by blocking with 5% BSA. Subsequently, the sections were incubated with primary antibodies at 4 °C overnight. Sections were incubated with the enhanced enzyme‐labelled sheep anti‐mouse/rabbit IgG polymer (PV9000; ZSGB‐BIO, China) at 37 °C for 20 min the following day. Images were obtained using a microscope (Nikon, Japan).

### Transmission Electron Microscopy

Renal cortical tissues were sectioned into one mm^3^ pieces and fixed in 2.5% glutaraldehyde. Following rinsing with 0.1 M phosphoric acid, the tissues were fixed with osmium tetroxide for 30 minutes, dehydrated, and embedded in acetone. The ultra‐thin sections (50–60 nm) were stained with 3% uranyl acetate and lead citrate, then captured the images by transmission electron microscopy (TEM) (JEM‐1400plus, Japan).

### Western Blot analysis

Murine renal tissues or HK‐2 cells were lysed in RIPA buffer supplemented with 1% phenylmethylsulfonyl fluoride (PMSF) and protease inhibitors. Protein concentration was determined using a BCA protein assay kit (Solarbio, China). Proteins were separated by SDS‐PAGE and transferred to a 0.45 µm PVDF membrane. After blocking with 5% skimmed milk at room temperature for one hour, the membrane was incubated with primary antibodies overnight at 4 °C. Following washes with TBST, the membrane was incubated with HRP‐conjugated secondary antibodies at room temperature for one hour and subsequently exposed to an ECL reagent (Millipore, USA) for detection using a Tanon 5200 imaging system (China). Quantitative analysis was performed using ImageJ software (NIH, USA). The primary antibodies used were in Table S2 (Supporting Information).

### Co‐Immunoprecipitation

Co‐immunoprecipitation by Pierce Protein A/G Magnetic Beads (Thermo Fisher Scientific, USA) was performed, incubated with anti‐ALDH2, anti‐FLAG, anti‐PHB2, anti‐SIRT3, or anti‐L‐Lactyl Lysine antibodies, and rotated at room temperature for one hour. To reduce non‐specific binding, cell lysates were pre‐cleared by incubation with protein A/G agarose beads for two hours with gentle rotation, followed by centrifugation at 1000 × g for five minutes at 4 °C. The pre‐cleared cell lysates were then added to the antibody‐bound magnetic beads and incubated for one hour at room temperature. The immune complexes were pelleted and washed three times with lysis buffer, resuspended in 1× SDS sample buffer, boiled, and subjected to SDS‐PAGE for subsequent Western blot analysis. The primary antibodies used were in Table S2 (Supporting Information).

### RNA Extraction and Quantitative Real‐Time PCR

Total RNA was extracted from cultured cells using TRIzol reagent (Invitrogen, USA) and reverse transcribed into cDNA using a kit (RR036A, Takara, Japan). Real‐time PCR (RT‐PCR) analysis was performed in triplicate using the SYBR Green PCR kit (RR820A, Takara, Japan) on a Bio‐Rad CFX PCR System (Bio‐Rad, USA). The primer sequences used were in Table S3 (Supporting Information).

### Immunofluorescence

The cells were washed three times with phosphate‐buffered saline (PBS), followed by incubation with 200 nM MitoTracker Deep Red (Beyotime Biotechnology) at 37 °C for 30 minutes. Afterward, the cells were fixed in 4% paraformaldehyde, blocked with 1% bovine serum albumin (BSA), and incubated overnight at 4 °C with primary antibodies (listed in Table S2, Supporting Information). After three washes with PBS, cells were incubated with fluorescence‐conjugated secondary antibodies (listed in Table S2, Supporting Information), followed by counterstaining of nuclei with DAPI. Images were captured using a confocal laser microscope (Nikon AXR, Japan).

### Measurement of Lactate

Lactate levels in serum, urine, and kidney tissue were quantified using a lactate detection kit (Promega, USA) following the instructions. The serum samples 500‐fold and urine samples 100‐fold were diluted. Kidney tissues were lysed with RIPA buffer, and the supernatant was used for lactate measurement. The lactate content in kidney tissues was normalized by tissue weight.

### ALDH2 Activity Measurement

ALDH2 activity was quantified using the ALDH2 Activity Assay (Abcam, UK) according to the manufacturer's instructions. Cells were collected, and the pellet was solubilized in extraction buffer, followed by centrifugation at 16000 × g at 4 °C for 20 minutes. The diluted sample was added to each well in a 96‐well plate and incubated at room temperature for three hours, followed by three washes. ALDH2 was extracted and its activity was assessed at 450 nm based on the absorbance value.

### Mitochondrial Membrane Potential and Mitochondrial Superoxide

The mitochondrial membrane potential (ΔΨm) and mitochondrial superoxide levels were assessed using the cationic fluorescent probes JC‐1 (Beyotime Biotechnology, China) and MitoSOX Red (Thermo Fisher Scientific, USA), respectively. Cells were washed twice with PBS, stained with JC‐1 or MitoSOX for 20 minutes at 37 °C, rinsed twice with binding buffer, and visualized using a Nikon AXR confocal laser microscope.

### Seahorse XFe96

The mitochondrial oxygen consumption rate (OCR) was assessed using a Seahorse XFe96 Flux Analyzer (Seahorse Biosciences, Agilent, USA) equipped with the Mito Stress Test kit. HK‐2 cells were seeded at a density of 2000 cells per well in XF96 plates. Following a 24‐hour incubation with either MA or medium, test compounds were sequentially administered: oligomycin (1.5 µM), FCCP (1.0 µM), and rotenone/antimycin A (both 0.5 µM). OCR measurements were averaged and normalized by the number of cells per well.

### Protein Half‐Life Assay

The cells transfected with Flag‐ALDH2 and Flag‐ALDH2‐K52R plasmids were treated with 100 µg mL^−1^ cycloheximide (MCE, USA) for 0, 2, 4, and 6 hours, collected the cell lysates to analyze PHB2 protein levels by Western blotting, and calculated the protein half‐life using GraphPad Prism 9.0 software.

### In Vivo Ubiquitination Assay

HEK‐293T cells were co‐transfected with HA‐tagged ubiquitin, Flag‐ALDH2, or Flag‐ALDH2‐K52R plasmids. After 48 hours, cells were harvested, and the cell lysates were immunoprecipitated with anti‐PHB2 antibody. Western blotting was then performed to detect ubiquitin protein levels.

### mRNA Sequencing

Total RNA was extracted from renal cortical tissues of MA‐AKI and control mice (n = 3), sequenced by the Novogene Bioinformatics Institute (Novogene, China) following standard protocols. The libraries were sequenced using the Illumina NovaSeq platform. Differential expression and enrichment analyses were using R software, with data visualization facilitated by the “ggplot2” package in R.

### Single‐Cell mRNA Sequencing

Freshly resected kidney samples from mice were minced and enzymatically digested to obtain single‐cell suspensions. Single‐cell libraries were prepared and sequenced by the Beijing Genomics Institute (BGI, China). Using the Seurat R package (version 4.0.4), The data were analyzed, including normalization, scaling, principal component analysis (PCA), and uniform manifold approximation and projection (UMAP) for dimension reduction and visualization of gene expression. Cell types were annotated based on canonical marker genes and differentially expressed genes (DEGs) identified by the FindAllMarkers function. Additionally, The Seurat AddModuleScore_UCell method was utilized to assess the glycolysis and lactylation activity in proximal tubule cells (PTCs).

### LC‐MS/MS Analysis

Cells transfected with Flag‐ALDH2 or control vector plasmids underwent immunoprecipitation (IP) – mass spectrometry (MS) analysis. The immunoprecipitation was carried out using an anti‐Flag antibody, and the proteins were then separated via SDS‐PAGE, followed by brilliant Coomassie blue staining. Bands corresponding to the ALDH2 protein were excised for subsequent identification of interacting proteins and lactylation sites via mass spectrometry (Jingjie PTM BioLab (HangZhou) Co., Inc.).

### Statistical Analysis

The statistical analyses with GraphPad Prism version 9.31 (GraphPad Software, Inc., La Jolla, CA) were conducted. Data were presented as mean ± SEM from three independent experiments. The normality of distribution and homogeneity of variance for all variables were verified using Shapiro‐Wilk and Levene tests, respectively. Statistical tests included Student's two‐tailed unpaired t‐test and one‐way or two‐way ANOVA followed by Tukey's test, depending on the experimental design involving one or two factors. Correlation analysis was conducted using Pearson's correlation coefficient. A *P*‐value of less than 0.05 was considered statistically significant.

### Ethics Approval and Consent to Participate

Serum and urine collections from patients were approved by the Ethics Committee of Peking Union Medical College Hospital (No. I‐22PJ856). All animal procedures were authorized by the PUMCH Institutional Ethics Committee of Animal Care and Use (No. XHDW‐2023‐171).

## Conflict of Interest

The authors declare no conflict of interest.

## Author Contributions

J.L. designed, performed the experiments and wrote the manuscript. X.S. and J.X. performed the experiments and data analysis. K.W. and F.H. helped conduct the animal studies. X.L., and L.C. supported this study technically. All authors contributed to the article and approved the submitted version.

## Supporting information



Supporting Information

## Data Availability

The data that support the findings of this study are available in the supplementary material of this article.
