# Supplementary material for: Aldehyde Dehydrogenase 2 Lactylation Aggravates Mitochondrial Dysfunction by Disrupting PHB2 Mediated Mitophagy in Acute Kidney Injury
Source: Adv Sci (Weinh). 2024 Dec 31;12(8):2411943. doi: 10.1002/advs.202411943 (PMC11848585; doi:10.1002/advs.202411943)
Supplement: Supplementary file 1 — Supporting Information [file ADVS-12-2411943-s001.docx]

**
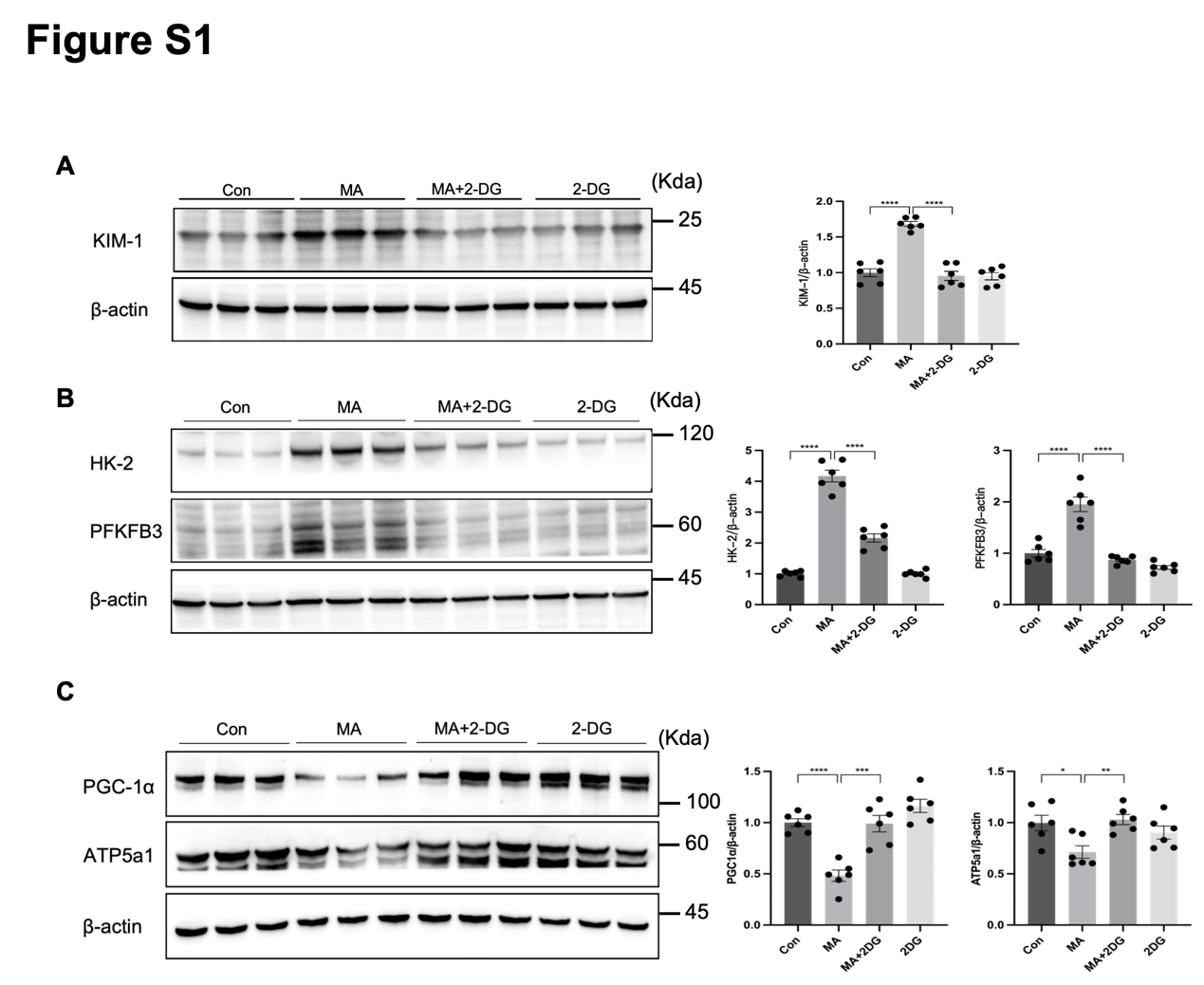
**

**Figure S1 2-DG inhibited glycolysis and alleviated mitochondrial dysfunction in MA-AKI mice.** (A-C) The expression of KIM-1, glycolysis-related enzymes (HK2 and PFKFB3), and mitochondria-related proteins (PGC-1α and ATP5a1) was measured by Western blotting in 2-DG-treated MA-AKI mice (n = 6). One-way ANOVA was used for the analysis. ^*^*P* < 0.05, ^**^ *P* < 0.01, ^***^ *P* < 0.001, ^****^ *P* < 0.0001. (Con, control; MA, maleic acid; 2-DG, 2-Deoxy-D-glucose; MA-AKI, maleic acid-induced AKI).


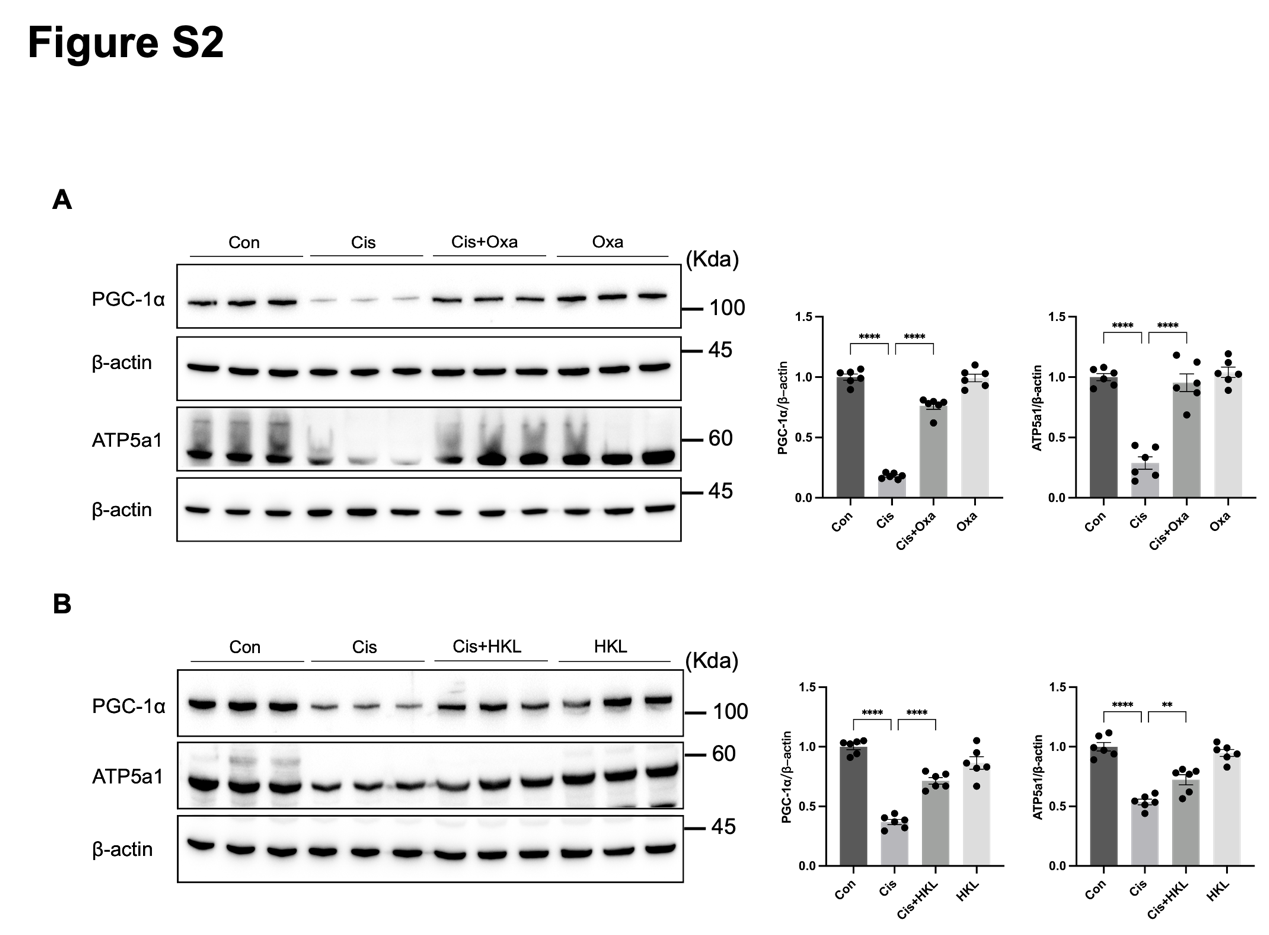


**Figure S2 Inhibition of lactate and Sirt3 activation attenuated cisplatin-induced mitochondrial dysfunction.** (A-B) The expression of mitochondria-related proteins (PGC-1α and ATP5a1) was measured by Western blotting in oxamate- and HKL pretreated AKI mice (n = 6). One-way ANOVA was used for the analysis. ^**^ *P* < 0.01, ^***^ *P* < 0.001, ^****^ *P* < 0.0001. (Con, control; Cis, cisplatin; Oxa, oxamate; HKL, honokiol).


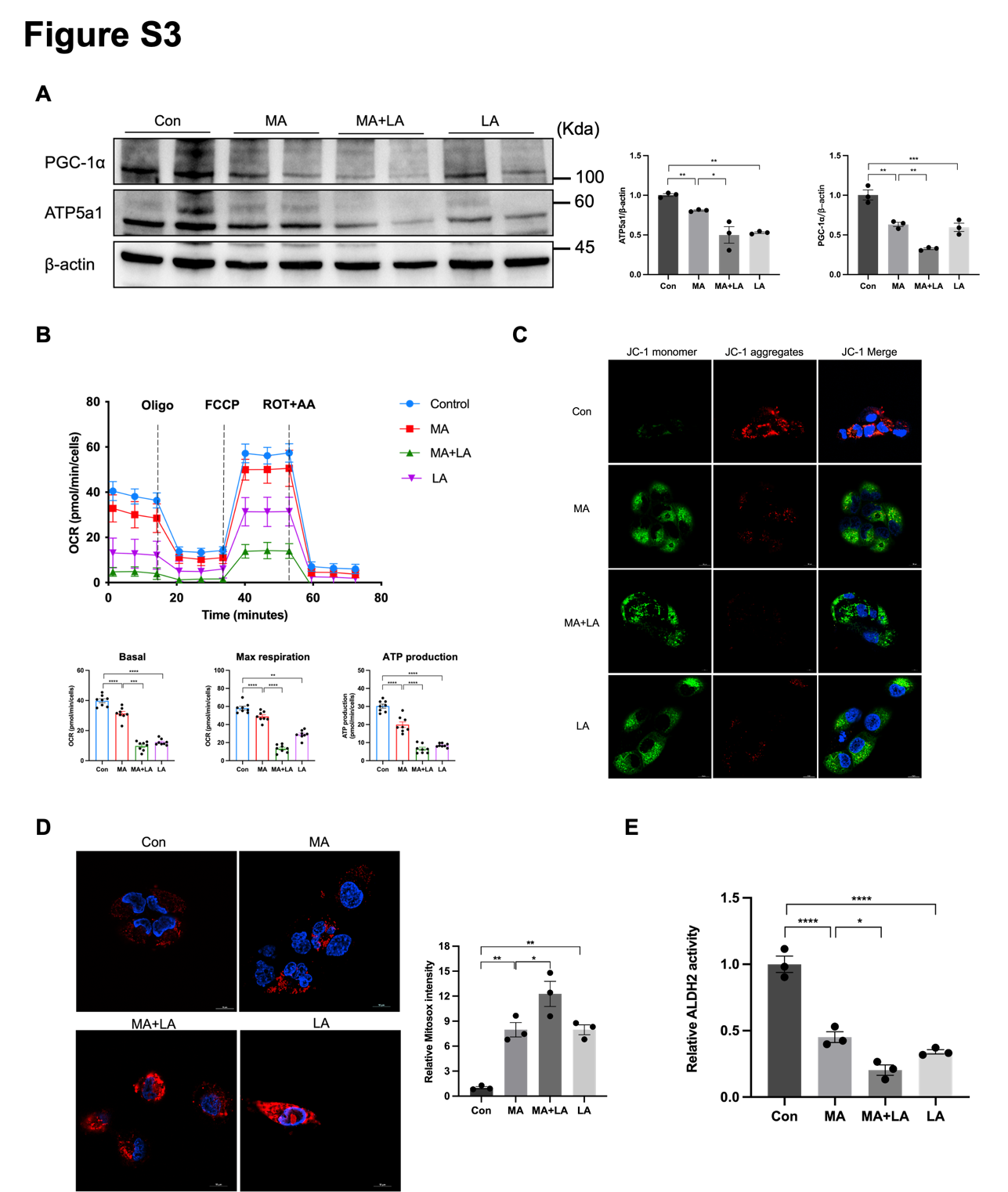


**Figure S3 Lactate aggravated mitochondrial dysfunction and inhibited ALDH2 activity in MA-induced HK-2 cells.** (A) The expression of mitochondria-related proteins (PGC-1α and ATP5a1) was measured by Western blotting in MA and lactate-stimulated HK-2 cells (n = 3). (B) ﻿Measurement of mitochondrial oxygen consumption ratio (OCR) in MA and lactate-stimulated HK-2 cells HK-2 cells (n = 8). (C) Images of JC-1 staining for mitochondrial membrane potential in four groups (n = 3). Scale bars, 20μm. (D) Images of MitoSox staining for mitochondrial superoxide in four groups (n = 3). Scale bars, 20μm. (E) ALDH2 activity was measured in four groups (n=3). One-way ANOVA was used for the analysis. ^*^*P* < 0.05, ^**^ *P* < 0.01, ^***^ *P* < 0.001, ^****^ *P* < 0.0001. (Con, control; MA, maleic acid; LA, lactate; OCR, oxygen consumption ratio; Oligo, oligomycin; ROT+AA, rotenone/antimycin A).


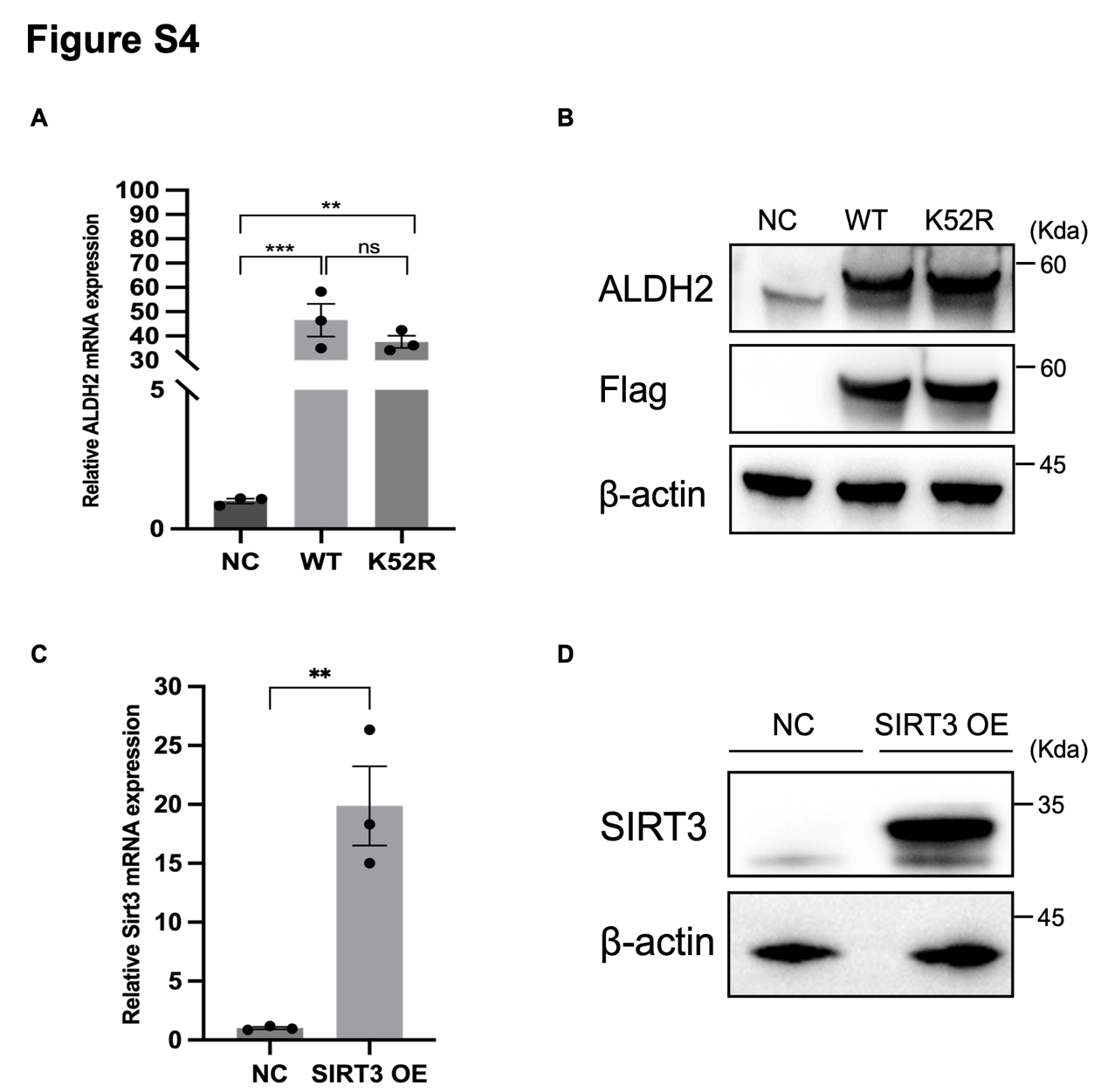


**Figure S4 The transfection efficiency of K52R and SIRT3 plasmid in HK-2 cells and effects on protein expression.** (A-B) In HK-2 cells transfected with NC, ALDH2-WT and ALDH2-K52R plasmids, ALDH2 mRNA and protein expression were measured by rt-PCR and Western blotting (n=3). (C-D) In HK-2 cells transfected with NC and SIRT3-overexpression plasmids, SIRT3 mRNA and protein expression were measured by rt-PCR and Western blotting (n=3). Unpaired student’s t-test and one-way ANOVA were used for the analysis. ^**^ *P* < 0.01, ^***^ *P* < 0.001; ns, not significant. (NC, normal control; WT, wild type; OE, overexpression;)


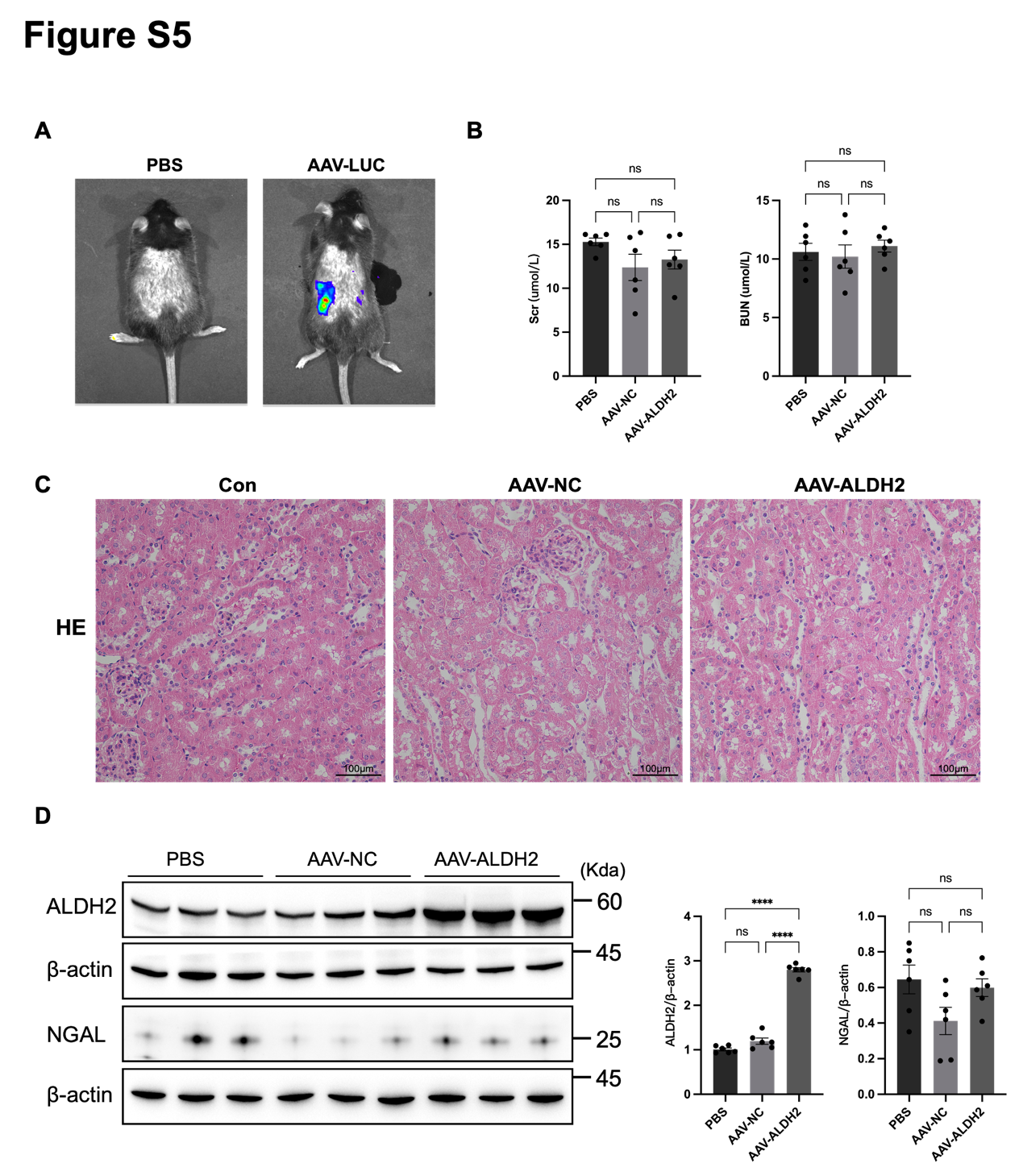


**Figure S5** **AAV infection was mainly in the left kidney and had no side effects on renal function.** (A) Luminescence was measured on day 28 in mice injected with PBS and AAV-LUC. (B) Serum creatinine (Scr) and urea nitrogen (BUN) levels in 3 groups (n = 6). (C) Images of hematoxylin-eosin (HE) staining (n = 6). Scale bars, 100μm. (D) The expression of ALDH2 and NGAL was measured by Western blotting (n=6). One-way ANOVA was used for the analysis. ^****^ *P* < 0.0001; ns, not significant. (Con, control; NC, normal control; AAV, adeno-associated viruses).

**
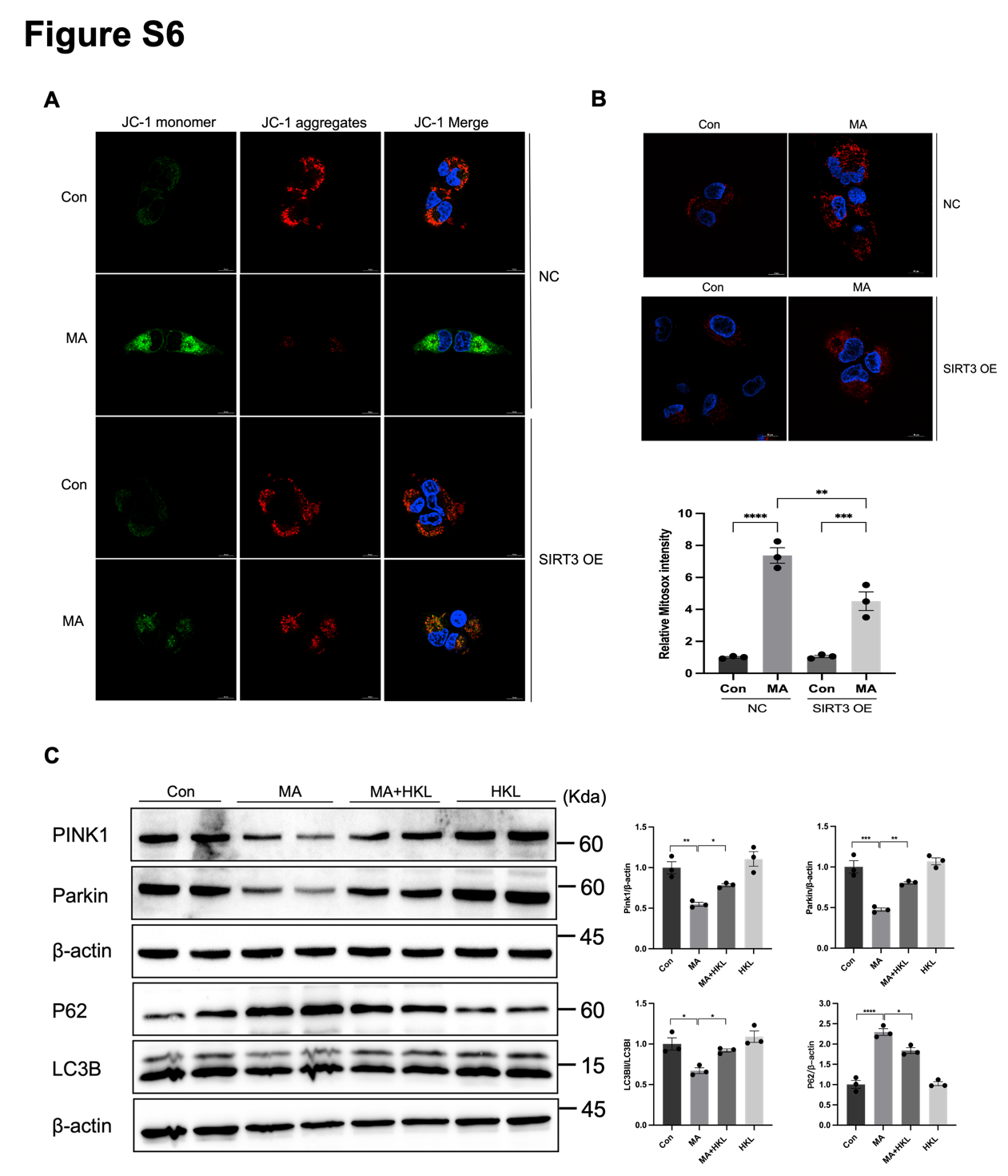
**

**Figure S6 Effect of SIRT3 on mitochondrial dysfunction and mitophagy in MA-stimulated HK-2 cells.** (A) Images of JC-1 staining for mitochondrial membrane potential in SIRT3-overexpressing HK-2 cells (n = 3). Scale bars, 20μm. (B) Images of MitoSox staining for mitochondrial superoxide in SIRT3-overexpressing HK-2 cells (n = 3). Scale bars, 20μm. (C) The expression of mitophagy-related proteins (Pink1, Parkin, LC3B and P62) was measured by Western blotting (n=3). One-way and two-way ANOVA were used for the analysis. ^*^*P* < 0.05, ^**^ *P* < 0.01, ^***^ *P* < 0.001, ^****^ *P* < 0.0001. (Con, control; MA, maleic acid; OE, overexpression; HKL, honokiol).


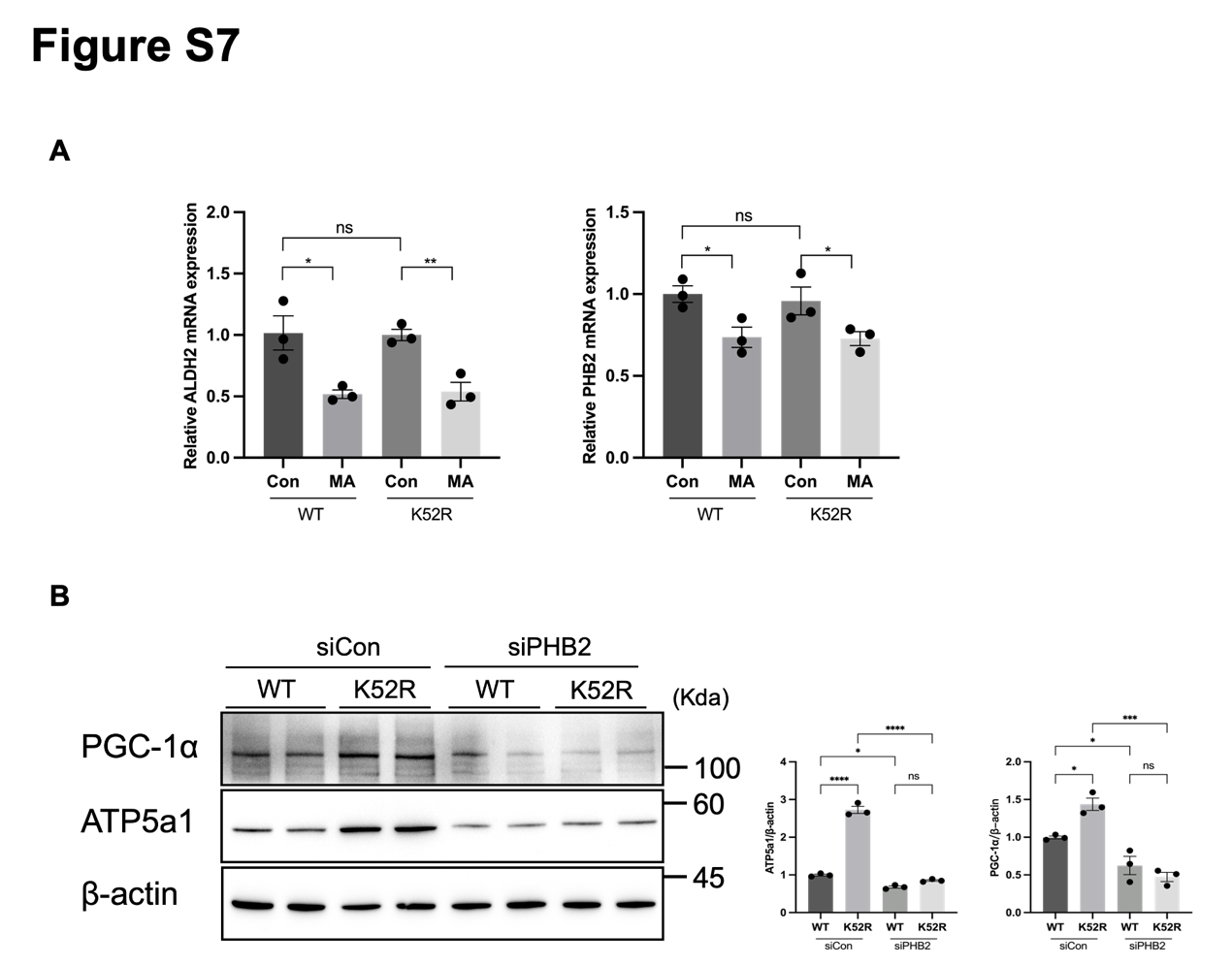


**Figure S7 Effects of ALDH2 lactylation on PHB2 mRNA expression and PHB2-mediated mitochondrial homeostasis.** (A) The mRNA expression of ALDH2 and PHB2 was measured by rt-PCR in HK-2 cells transfected with ALDH2-WT and ALDH2-K52R plasmids (n=3). (B) The expression of mitochondria-related proteins (PGC-1α and ATP5a1) was measured by Western blotting in siPHB2 and siCon groups (n=3). Two-way ANOVA was used for the analysis. ^*^*P* < 0.05, ^**^ *P* < 0.01, ^***^ *P* < 0.001, ^****^ *P* < 0.0001; ns, not significant. (Con, control; MA, maleic acid; siCon, control siRNA; siPHB2, PHB2 siRNA)

**Table S1 Clinical characteristics of AKI and minor lesions patients.**

| Clinical data | Control (n=12) | AKI (n=12) |
| --- | --- | --- |
| Male (%) | 6 (50.0) | 7 (58.3) |
| Age (years) | 46.3±11.3 | 48.5±17.6 |
| Hemoglobin (g/l) | 144.80±17.42 | 122.20±39.66 |
| Albumin (g/l) | 40.00±8.43 | 40.75±3.57 |
| Scr (umol/l) | 78.00±20.98 | 226.30±73.99 |
| eGFR (ml/(min*1.73m^2^)) | 94.9±17.38 | 28.33±11.12 |
| K (mmol/l) | 3.84±0.44 | 3.71±0.40 |
| Ca (mmol/l) | 2.26±0.15 | 2.32±0.13 |
| P (mmol/l) | 1.18±0.10 | 1.22±0.38 |
| UA (μmol/L) | 377.30±76.77 | 329.60±141.70 |
| LDH (U/L) | 178.80±32.74 | 195.50±46.62 |
| TCO2 (mmol/l) | 25.52±2.76 | 23.88±4.14 |

Note: Scr, serum creatinine; eGFR, estimated glomerular filtration rate.

**Table S2 Primary antibodies used in this study.**

| **Antibody** | **Source** | **Catalog** |
| --- | --- | --- |
| ALDH2 | Abcam | ab227021 |
| L-Lactyl Lysine | Jingjie PTM BioLab | PTM-1401 |
| Acetyllysine | Jingjie PTM BioLab | PTM-101 |
| KIM-1 | Abcam | ab78494 |
| NGAL | Abcam | ab216462 |
| PGC-1α | ABclonal | A12348 |
| ATP5a1 | Proteintech | 14676-1-AP |
| HK2 | Abcam | ab209847 |
| PFKFB3 | Abcam | ab181861 |
| PKM2 | CST | #4053 |
| SIRT3 | Proteintech | 10099-1-AP |
| SIRT1 | Abcam | ab110304 |
| PHB2 | Proteintech | 12295-1-AP |
| PINK1 | Proteintech | 23274-1-AP |
| Parkin | Proteintech | 14060-1-AP |
| LC3B | Abcam | ab192890 |
| P62 | Abcam | ab109012 |
| HA-Tag | Proteintech | 81290-1-RR |
| FLAG-Tag | Proteintech | 66008-4-Ig |
| β-actin | ABclonal | AC038 |

**Table S3 Primers used for real-time PCR analysis.**

| **Gene** | **Sequence 5’-3’** | **Species** |
| --- | --- | --- |
| ALDH2 | F: CCATTGACGGAGACTTCTT  R: GCTACCTTCATCACAACCA | Human |
| PHB2 | F: CTGGACTACGAGGAACGA  R: GCTGTGAGGCATTGAACT | Human |
| SIRT3 | F: ACTCCCATTCTTCTTTCACA  R: CGATGTTCTGCGTGTAGA | Human |
| β-actin | F: GGACCTGACTGACTACCT  R: CTTAATGTCACGCACGATT | Human |

**Table S4 Candidate interacting proteins of ALDH2 identified by mass spectrometry**

| **Accession** | **Protein** | **MW(KDa)** | **Coverage(%)** | **Unique Peptides** |
| --- | --- | --- | --- | --- |
| Q04695 | KRT17 | 48.1 | 53 | 10 |
| Q13642 | FHL1 | 36.2 | 49 | 16 |
| Q14103 | HNRNPD | 38.4 | 45 | 25 |
| O75323 | NIPSNAP2 | 33.7 | 45 | 13 |
| P55084 | HADHB | 51.3 | 45 | 14 |
| P36957 | DLST | 48.7 | 40 | 14 |
| **Q99623** | **PHB2** | **33.3** | **39** | **9** |
| Q14247 | CTTN | 61.5 | 38 | 25 |
| P48735 | IDH2 | 50.9 | 38 | 12 |
| P17931 | LGALS3 | 26.1 | 37 | 9 |
| Q13643 | FHL3 | 31.2 | 35 | 8 |
| P25311 | AZGP1 | 34.2 | 35 | 8 |
| O75688 | PPM1B | 52.6 | 33 | 12 |
| O43148 | RNMT | 54.8 | 33 | 13 |
| P31483 | TIA1 | 42.9 | 33 | 6 |
| P05089 | ARG1 | 34.7 | 33 | 6 |
| Q9H2U1 | DHX36 | 114.7 | 32 | 21 |
| P10515 | DLAT | 69 | 32 | 17 |
| P09543 | CNP | 47.5 | 32 | 9 |
| Q15424 | SAFB | 102.6 | 31 | 23 |
| P31025 | LCN1 | 19.2 | 31 | 6 |
| Q9UGN5 | PARP2 | 66.2 | 30 | 16 |
| Q9UGI8 | TES | 48 | 29 | 11 |
| P25705 | ATP5F1A | 59.7 | 27 | 12 |
| Q15942 | ZYX | 61.2 | 19 | 7 |
| Q5BKZ1 | ZNF326 | 65.6 | 19 | 9 |
| Q8NHP8 | PLBD2 | 65.4 | 19 | 11 |
| O00330 | PDHX | 54.1 | 19 | 9 |
| P00367 | GLUD1 | 61.4 | 19 | 8 |
| P07339 | CTSD | 44.5 | 19 | 6 |
